# Schistosomiasis in Africa: An Emerging Tragedy in Our New Global Health Decade

**DOI:** 10.1371/journal.pntd.0000485

**Published:** 2009-09-29

**Authors:** Peter J. Hotez, Alan Fenwick

**Affiliations:** 1 Department of Microbiology, Immunology, and Tropical Medicine, The George Washington University, Washington, D. C., United States of America; 2 Sabin Vaccine Institute, Washington, D. C., United States of America; 3 Schistosomiasis Control Initiative, Imperial College London, London, United Kingdom

*Despite new information that the disease burden of schistosomiasis in Africa may be equivalent to malaria or HIV/AIDS and a simple annual anthelminthic treatment for this disease is available for less than 50 cents per person including delivery costs, we now know that fewer than 5% of the infected population is receiving coverage. To date, this situation represents one of the first great failures of the “global health decade” that began in 2000.*

Although it has not been officially labeled as such, there are many good reasons to consider the first years of the 21st century as the global health decade [1]. Through the President's Emergency Program for AIDS Relief (PEPFAR), the President's Malaria Initiative (PMI), the Global Fund to Fight AIDS, Tuberculosis, and Malaria, and the sprouting of numerous global health advocacy organizations, tens of billions of dollars have been committed so far for HIV/AIDS, tuberculosis, and malaria, i.e., the three major killer infections of humankind, with millions of people now placed on treatment for these conditions. Over the same period, there have even been some impressive results for providing preventive chemotherapy treatments for some of the major neglected tropical diseases (NTDs). In 2007, an estimated 546 million people received anthelminthic treatments for lymphatic filariasis (LF), or approximately 42% of the population at risk, while in 2005, 46% of eligible populations received ivermectin treatments for onchocerciasis (river blindness) [2]. With the active involvement of global partnerships for LF and onchocerciasis, including the Global Programme to Eliminate LF (GPELF), the African Programme for Onchocerciasis Control (APOC), and the Organization to Eliminate Onchocerciasis in the Americas (OEPA), together with ongoing donations from GlaxoSmithKline and Merck & Co., Inc. to provide albendazole and ivermectin, respectively, there is great optimism that coverage for these conditions will continue to increase, and that eventually these great scourges will some day be eliminated as public health problems. Two other NTDs, namely leprosy and human African trypanosomiasis, are also being targeted for elimination.

Unfortunately, other NTDs have not fared so well in terms of coverage. Today it is believed that fewer than 10% of eligible populations living in endemic regions of Africa, Asia, and the Americas are receiving annual treatments for their schistosomiasis, intestinal helminth infections, and/or trachoma [2]. The World Health Organization (WHO) and several leading public private partnerships and non-governmental development organizations are actively working to correct this situation and to steadily increase global coverage to the levels of LF and onchocerciasis. Of these, we believe that the single largest gap in mass drug administration for a serious NTD has to be the almost non-existent global coverage for schistosomiasis.

There are an estimated 207 million people infected with one of the major schistosomes [3], with more than 90% of the cases occurring in sub-Saharan Africa [3],[4]. Through a full consideration of the amount of end-organ pathologies to the liver (in the case of *Schistosoma mansoni* and *S. japonicum* infections), and to the bladder and kidneys (in the case of *S. haematobium* infection) [5], together with the chronic morbidities associated with impaired child growth and development, chronic inflammation, anemia, and other nutritional deficiencies, some new disease burden assessments estimate that schistosmiasis accounts for up to 70 million disability-adjusted life years (DALYs) lost annually [6]. This global burden estimate exceeds that of malaria or tuberculosis, and is almost equivalent to the DALYs lost from HIV/AIDS [6]. Further, almost 300,000 people die annually from schistosomiasis in Africa [5], and there is evidence that female genital schistosomiasis caused by *S. haematobium* may significantly increase the likelihood of contracting HIV/AIDS [7].

Paired with these devastating health statistics is the equally alarming finding that fewer than 5% of the world's people with schistosomiasis are today receiving praziquantel, a specific anthelminthic treatment that costs as little as 8 cents per tablet [2]. At the April 2009 NTD Science and Technical Advisory Group (STAG) meetings in Geneva, the WHO reported that compiled statistics given to them by the member countries confirmed that fewer than 5% of the estimated people infected with schistosomiasis actually received treatment in 2008 (L. Savioli, personal communication). In Burkina Faso, annual treatment for a child with praziquantel (including delivery) has been shown to cost 32 cents [8]. Annual or every other year treatment with praziquantel has been shown to improve child growth and development, and to reverse the anemia and some of the end-organ pathologies linked to schistosomiasis [9],[10]. With support from the Bill & Melinda Gates Foundation, the Schistosomiasis Control Initiative (SCI) was created in 2002 in order to promote increased access to praziquantel in the at-risk areas of sub-Saharan Africa [11]. Working in collaboration with health ministries in six countries, an estimated 13, 9, and 8 million school-aged children received praziquantel treatments in 2005, 2006, and 2007, respectively [2],[12]. In so doing, SCI has delivered most of the praziquantel to national programs providing African coverage, supplemented by drug donations from MedPharm, a generic pharmaceutical company that utilizes donations from Canadian citizens. However, an expansion of these activities in order to target most of the at-risk populations in sub-Saharan Africa would require a dramatic increase in donations for drugs and delivery costs if we are to begin approaching the levels of coverage that currently benefit global populations at risk for LF and onchocerciasis. A more recent (2009) grant from the Gates Foundation to the Global Network for Neglected Tropical Diseases will mobilize resources for expanding preventive chemotherapy treatments [13], which should help promote improvements in praziquantel coverage, but there are still important hurdles that first must be urgently overcome.

*Availability of praziquantel*. Throughout much of sub-Saharan Africa there is insufficient praziquantel available for treatment. Praziquantel dosage is one tablet per 15 kg of body mass, so on average praziquantel tablet costs are only 20 cents per child and 24–32 cents per adult dose, depending on weight. However, because schistsomaisis is so widespread and the major control strategy relies on mass drug administration to either school-aged children or total populations, even this modest price exceeds the health budget of many disease-endemic countries. In 2008, Merck KgaA (headquartered in Darmstadt, Germany) made an important first step to ameliorate this situation by agreeing to donate 200 million tablets of praziquantel over the next decade, but this donation still represents a modest fraction of the drug required because 20 million tablets per year would treat just eight million children. According to a recent WHO stakeholder meeting on access to essential medicines for NTDs (Thematic Stakeholder Meeting on Access to Essential Medicines for Neglected Tropical Diseases, at International Conference Centre Geneva, Switzerland, 23–25 March 2009), the United States Agency for International Development (USAID) has also donated praziquantel for schistosomiasis control in six endemic countries through their NTD control program, with additional donations anticipated by the British Department for International Development (DFID). Our recommendation is that approximately US$100 million annually be made available to purchase the 1,200 million praziquantel tablets required each year, for at least 5 years. With these resources, sufficient praziquantel would be made available to treat approximately 400 million people annually (or in some cases every other year) who are either actively infected with schistosomes or who are considered at high risk for acquiring the infection. In addition to donations by Merck KgaA, USAID, and DFID, efforts are being made to reach this target by the resource mobilization arm of the Global Network for Neglected Tropical Diseases working together with the WHO. Other members of the G8 (Group of Eight countries) may also be mobilized, with a possible commitment at an annual G8 summit within the next 2 years. Once funds are committed, it will be necessary to ensure adequate manufacturing capacity by coordinating increased production of the active pharmaceutical ingredient (API), now mostly done in Asia, and then formulation into tablets now mostly conducted either in India or South Korea, or at a Merck KgaA–owned plant in Mexico.

*Praziquantel distribution*. Schistosomiasis exhibits a focal distribution, and the symptoms are often difficult to recognize both by the individuals infected and by health personnel who normally staff the primary health care facilities in rural Africa. Additionally, the early stages of schistosomiasis, when treatment is most beneficial, often show only mild yet debilitating symptoms, which lead to serious consequences later in life. This means that praziquantel cannot be delivered in the way that many drugs are distributed, i.e., by sending them routinely to the Central Medical Stores and then via districts to health units. To do so would result in inefficient and fragmented use of the drugs because the rural poor infected with schistosomiasis rarely seek treatment for their early stage symptoms, and diagnostic testing is often not performed. The best way forward for schistosomiasis control then is for mass drug administration to be delivered to total at-risk populations defined where a survey shows prevalence of over 50% in school-aged children (although pre-school aged children can also be infected), and mass drug administration to children aged 6–16 years where survey prevalence is between 10% and 50% [14]. This regimen would be repeated until prevalence falls below the above thresholds. The timeline strategy recommended by SCI (working with WHO) is therefore to carry out the mapping of schistosomiasis distribution with an assessment of prevalence, conduct evidence-based advocacy to decision makers, determine drug needs, order drugs, and finally train health ministry staff, teachers, and community drug volunteers in order to deliver the safe and effective drugs, receive drugs through the port of entry, and/or distribute the drugs to rural centers, followed by mass drug distribution. Treatments are then repeated annually in areas of high endemicity and sometimes every 2 years in areas of lower endemicity. In terms of costs, we recommend that drug tablet costs should be accompanied by a budget of approximately 25 cents for delivery, including advocacy, training, and monitoring and evaluation. Thus, in addition to the ▒100 million for praziquantel costs as outlined above, an additional ▒100 million is also required annually to pay for the essential delivery costs. Used appropriately and when possible integrated with other NTD control efforts [2],[13], such funds could dramatically reduce schistosome-related morbidity in Africa.

*Operational research*. Mass drug administration using praziquantel on a large scale raises many operational research questions, and all control initiatives should be accompanied by a budget for operational research. The range of questions that need to be addressed include actual costs, drug efficacy, monitoring for possible development of drug resistance, recording compliance, measuring clinical improvement after treatment through ultrasound and other technologies, and other clinical improvements, including growth in children and reductions in anemia. Thereafter, the optimal use of praziquantel will need to be determined, including frequency of treatment (annually or every 2 years), and then an exit strategy once prevalence and intensity are reduced [15]. Some of these activities and others are soon to begin, funded by the Gates Foundation through the Schistosomiasis Consortium for Operational Research and Evaluation (SCORE) (D. G. Colley, personal communication).

*Back-up control tools*. There is currently no strong evidence for the emergence of praziquantel resistance against schistosomes. However, if schistosomiasis control activities are scaled and over one hundred millions of at-risk populations receive praziquantel, it would be wise to consider this possibility and look into the development of a second anthelminthic drug with an equivalent target product profile [16]–[18]. Such upstream activities should be encouraged under the auspices of the Special Programme for Research and Training in Tropical Diseases (TDR), possibly in collaboration with interested pharmaceutical companies committed to orphan drug development and major donors such as the Gates Foundation. First generation anti-schistosome recombinant vaccines are under development for *S. mansoni* infection (by both the Sabin Vaccine Institute and several Brazilian institutions, including the Oswaldo Cruz Foundation, Federal University of Minas Gerais, and Instituto Butantan) and *S. haematobium* infection (by the Institut Pasteur in Lille, France) [19],[20]. Such vaccines could be used together with praziquantel in a combined and integrated program of vaccine-linked chemotherapy [21].

The scaling up of praziquantel manufacture and distribution, together with operational research and new product research and development, would represent a global assault on schistosomiasis. Such activities could reduce one of Africa's greatest disease burdens [4], but at a cost far lower than that associated with better known conditions such as HIV/AIDS and malaria [13]. Accordingly, global health policy makers, major donors (including the bilaterals and the Gates Foundation), the WHO, and the major public–private partnerships devoted to schistosomiasis control (including SCI and the Global Network for Neglected Tropical Diseases) are moving quickly to increase access to these low-cost, effective treatments in order to achieve this goal. If successful, we could avoid a tragic oversight and grasp an extraordinary opportunity to help the poorest people in Africa in the context of an otherwise successful decade of global health. In 2001, the World Health Assembly adopted a resolution that set as a global target the regular treatment of at least 75% of school-aged children at risk for both soil-transmitted helminth infections and schistosomiasis by 2010 [22]. Although it is clear that this target will not be met, it should serve as a wake-up call to increase our efforts in Africa for schistosomiasis control. There is still time to consign this disease to history [23].
